# MORC Proteins: Novel Players in Plant and Animal Health

**DOI:** 10.3389/fpls.2017.01720

**Published:** 2017-10-18

**Authors:** Aline Koch, Hong-Gu Kang, Jens Steinbrenner, D'Maris A. Dempsey, Daniel F. Klessig, Karl-Heinz Kogel

**Affiliations:** ^1^Centre for BioSystems, Land Use and Nutrition, Institute for Phytopathology, Justus Liebig University Giessen, Giessen, Germany; ^2^Department of Biology, Texas State University, San Marcos, TX, United States; ^3^Boyce Thompson Institute for Plant Research, Ithaca, NY, United States

**Keywords:** plant MORCs, human MORCs, transcriptional gene silencing, RNA interference (RNAi), RNA-directed DNA methylation, immunity, pathogen

## Abstract

Microrchidia (MORC) proteins comprise a family of proteins that have been identified in prokaryotes and eukaryotes. They are defined by two hallmark domains: a GHKL-type ATPase and an S5 fold. MORC proteins in plants were first discovered via a genetic screen for Arabidopsis mutants compromised for resistance to a viral pathogen. Subsequent studies expanded their role in plant immunity and revealed their involvement in gene silencing and transposable element repression. Emerging data suggest that MORC proteins also participate in pathogen-induced chromatin remodeling and epigenetic gene regulation. In addition, biochemical analyses recently demonstrated that plant MORCs have topoisomerase II (topo II)-like DNA modifying activities that may be important for their function. Interestingly, animal MORC proteins exhibit many parallels with their plant counterparts, as they have been implicated in disease development and gene silencing. In addition, human MORCs, like plant MORCs, bind salicylic acid and this inhibits some of their topo II-like activities. In this review, we will focus primarily on plant MORCs, although relevant comparisons with animal MORCs will be provided.

## The discovery of MORC proteins

**Figure 1:**
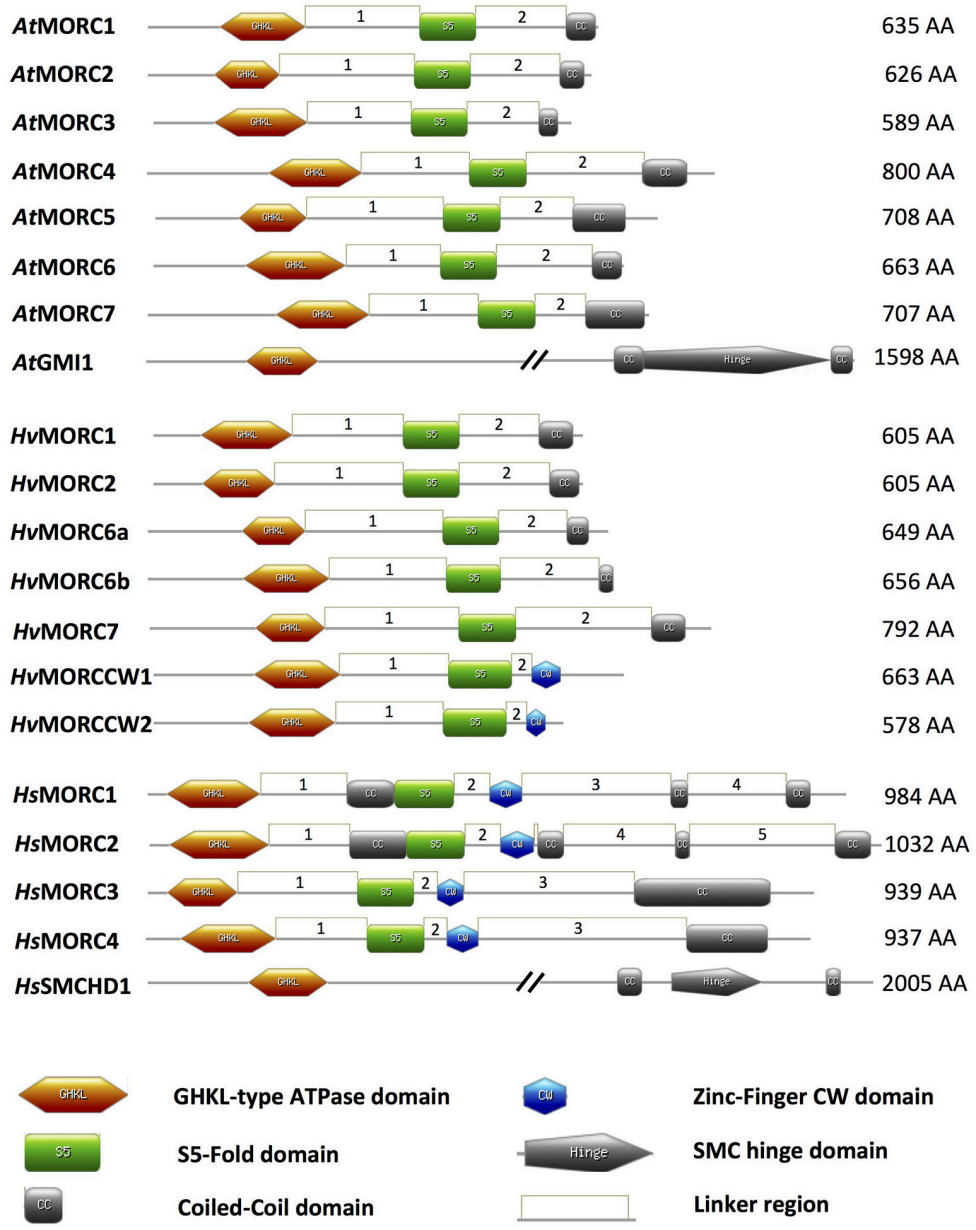
Schematic of MORC architecture. Domain organization of MORC family members from *Arabidopsis thaliana* (*At*), *Hordeum vulgare* (*Hv*), and *Homo sapiens* (*Hs*) are shown. In plant and animal MORC proteins, the N-terminal region contains a highly conserved GHKL-ATPase domain combined with an S5 fold domain. These domains are connected via an unstructured region or linker (L1). In addition to the N-terminal GHKL-ATPase module, many plant MORC proteins contain a coiled-coil (CC) domain at their C-terminal region that is connected via a linker (L2) to the S5 fold domain. Other unstructured regions or linkers connecting different structured domains are depicted as linkers 3–5 (L3–L5). Many animal MORC proteins and some plant MORC proteins also carry a zinc-finger CW domain in the C-terminal region of the protein. Some animal, but not plant, MORC proteins carry an additional CC domain between the GHKL-ATPase domain and the S5 fold domain. Protein domains were drawn using MyDomains—Image creator (http://prosite.expasy.org/mydomains/). Note all protein domain structures have been drawn to scale except *At*GMI1 and *Hs*SMCHD1.

MORC proteins were initially identified in mice. An insertional mutation in a gene encoding a 108 kDa nuclear protein involved in male primordial germ cell development caused complete arrest of spermatogenesis at an early point in meiotic prophase (Watson et al., 1998; Inoue et al., 1999). Compromised gene function resulted in extensive germ cell apoptosis leading to aberrant testes, which led to the name *morc* (for *microrchidia*), a medical term for abnormally small testes (Watson et al., 1998). The first report of a plant MORC came in 2008 from Kang and co-workers, who were using a forward genetic screen to identify components involved in immune signaling in the small flowering plant *Arabidopsis thaliana* (commonly known as thale cress or mouse-ear cress) (Kang et al., 2008). In Arabidopsis, resistance to infection by TCV (TURNIP CRINKLE VIRUS) is governed by HRT (HYPERSENSITIVE RESPONSE TO TCV), a CC (coiled coil)-NB (nucleotide binding)-LRR (leucine rich repeat)-type resistance (R) protein. When HRT detects the presence of the TCV coat protein (CP), it triggers the activation of plant immune responses, including increased defense gene expression, accumulation of the defense hormone salicylic acid (SA), and development of a hypersensitive response (HR), a form of programmed cell death that occurs at the site(s) of pathogen entry (Kang et al., 2008). *At*MORC1 (then named *At*CRT1 for COMPROMISED RECOGNITION OF TCV) was identified as a component of the HRT signaling pathway since ethyl methanesulfonate-generated mutants defective for *At*MORC1 survived expression of a TCV CP transgene that elicited lethal necrosis in the parental transgenic line. Sequence analysis subsequently revealed that *At*MORC1 contains the characteristic domains of MORC proteins (Iyer et al., 2008; Figure 1).

## The structure of animal and plant MORCs

MORC proteins comprise a subset of the GHKL ATPase superfamily for which DNA Gyrase, HSP90 (Heat Shock Protein 90), Histidine Kinase, and DNA mismatch repair protein MutL (mutator L) serve as prototypic members. MORC proteins are widely distributed in eukaryotic species; they also are present in prokaryotes, although their distribution is sporadic (Iyer et al., 2008). The hallmark of MORC proteins is a highly conserved tripartite GHKL ATPase/kinase domain containing a Bergerat ATP binding fold at the amino (N)-terminus coupled to an S5 fold domain containing a MORC-specific motif (Iyer et al., 2008) via an unstructured region or linker designated L1 (Figure 1; Dutta and Inouye, 2000; Li et al., 2013). The combined GHKL-S5 domains constitute an active “GHKL-type ATPase” module involved in ATP binding and hydrolysis; these activities have been demonstrated for many GHKL superfamily members in prokaryotes and mammals (Ban and Yang, 1998; Smith and Maxwell, 1998; Hu et al., 2003; Meyer et al., 2003; Corbett and Berger, 2005), and for MORC proteins identified in several plant species, such as barley, Arabidopsis, potato, tomato, and tobacco (Kang et al., 2008; Lorković et al., 2012; Langen et al., 2014; Manosalva et al., 2015; Manohar et al., 2017).

While comparison of human and plant MORC amino acid (aa) sequences reveals some conservation in their N-terminal portions, particularly in the tripartite GHKL ATPase and S5 fold domains, there is little conservation in their carboxy (C)-terminal portions (Manohar et al., 2017; Figure 1). However, the majority of MORC proteins identified in plants and vertebrates are predicted to contain a CC domain at their C-terminus (Inoue et al., 1999; Perry and Zhao, 2003; Li et al., 2013; Langen et al., 2014; Manosalva et al., 2015; Figure 1). Additional CC domains further upstream in the N-terminal region also have been identified in some animal, but not plant, MORC proteins (Figure 1). CC domains have been shown to play critical roles in regulating protein-protein and protein-DNA interactions, as well as influencing protein stability and subcellular location (Burkhard et al., 2001; Lupas and Gruber, 2005; Li et al., 2013). The function of the internal CC domains is currently unknown, but the combined observations that (i) many members of the GHKL ATPase superfamily form dimers via their diverse C-terminal dimerization domains (Dutta and Inouye, 2000), and (ii) deletion of the C-terminal CC region suppresses homo-dimerization of human *Hs*MORC3 *in vivo* (Mimura et al., 2010) and homo-dimerization of recombinant tomato and potato MORC1 *in vitro* (Manosalva et al., 2015), suggests that the C-terminal CC domain is important for protein-protein interactions. Interestingly, the CC-containing C-terminal regions of tomato and potato MORC1 are phosphorylated by a *Nicotiana benthamiana* protein extract *in vitro* and can induce spontaneous cell death when transiently expressed in *N. benthamiana*. These findings further suggest that this region plays a key role in regulating MORC1 activity (Manosalva et al., 2015).

Another domain that is found in some MORC proteins is the zinc finger (zf)-CW domain (Li et al., 2013; Figure 1). This domain, which has been identified in various chromatin-related factors, consists of four conserved cysteines (C) and two to four position-conserved tryptophans (W) in a motif of ~60 residues (Perry and Zhao, 2003). CW domains from several different proteins, including some human *Hs*MORCs, have been shown to bind the N-terminal tail of histone H3 with varying affinity depending on the methylation state of lysine 4 (He et al., 2010; Hoppmann et al., 2011). Thus, CW-containing proteins are proposed to play important roles in regulating chromatin structure and epigenetic memory. In animal MORC proteins, the CW domain is present upstream of the C-terminal CC domain (Perry and Zhao, 2003; Li et al., 2013; Hong et al., 2017; Figure 1). By contrast, only a minority of plant MORCs contain a CW domain, and it is located at the C-terminus in place of a CC-domain (Langen et al., 2014).

A divergent group of MORC proteins containing a C-terminal SMC (STRUCTURAL MAINTENANCE OF CHROMOSOMES) hinge domain coupled to the N-terminal GHKL-S5 domain also has been identified in animals and plants (Blewitt et al., 2008; Böhmdorfer et al., 2011; Leong et al., 2013). Other members of the SMC protein superfamily are chromosomal ATPases that play key roles in various aspects of higher-order chromosome organization and dynamics (Losada and Hirano, 2005). SMC proteins are highly conserved from prokaryotes to eukaryotes. They contain an ATPase module (although the module present in MORCs differs from those of other SMC proteins) and an SMC hinge domain, which mediates protein dimerization and DNA binding (Figure 1).

## The MORC family contains multiple members in plants and mammals

The MORC family in Arabidopsis is currently composed of eight members. Seven share a similar structure consisting of an N-terminal GHKL ATPase module linked to a C-terminal CC domain: *At*MORC1 (At4g36290, syn. CRT1), *At*MORC2 (At4g36280, syn. CRH1), *At*MORC3 (At4g36270, syn. CRH2), *At*MORC4 (At5g50780, syn. CRH4), *At*MORC5 (At5g13130, syn. CRH5), *At*MORC6 (At1g19100, syn. CRH6, DEFECTIVE IN MERISTEM SILENCING 11 [DMS11]), and *At*MORC7 (At4g24970, syn. CRH3) (Figure 1; Table 1; Kang et al., 2008; Lorković et al., 2012; Moissiard et al., 2014). Phylogenetic analyses revealed that *At*MORC1, *At*MORC2, and *At*MORC3 are the most closely related, exhibiting 75–81% sequence identity at the aa level; thus, they were assigned to clade I of the MORC phylogenetic tree (Langen et al., 2014). The other Arabidopsis proteins share less than 50% aa identity with *At*MORC1 (Kang et al., 2008) and were assigned to clade II (*At*MORC4, *At*MORC5, and *At*MORC7) and clade III (*At*MORC6) (Kang et al., 2008; Langen et al., 2014). In comparison to these proteins, another potential member of the Arabidopsis MORC family, designated GMI1 (γ-IRRADIATION AND MITOMYCIN C INDUCED 1), contains a C-terminal SMC hinge domain coupled to the GHKL ATPase module (Figure 1; Böhmdorfer et al., 2011).

**Table 1:** MORC homologs in different eukaryotic species.

**Organism**	**MORC homologs**	**References**
*Arabidopsis thaliana*	*At*MORC1, *At*MORC2, *At*MORC3, *At*MORC4, *At*MORC5, *At*MORC6, *At*MORC7 *At*GMI1	Kang et al., 2008; Böhmdorfer et al., 2011
*Hordeum vulgare*	*Hv*MORC1, *Hv*MORC2, *Hv*MORC6a, *Hv*MORC6b, *Hv*MORC7 *Hv*MORCCW1, *Hv*MORCCW2	Langen et al., 2014; Mascher et al., 2017
*Solanum lycopersicum*	*Sl*MORC1, *Sl*MORC3, *Sl*MORC4a, *Sl*MORC4b, *Sl*MORC6a, *Sl*MORC6b, *Sl*MORC7	Langen et al., 2014; Manosalva et al., 2015
*Solanum tuberosum*	*St*MORC1, *St*MORC4a, *St*MORC4b, *St*MORC6a, *St*MORC6b, *St*MORC7	Manosalva et al., 2015
*Nicotiana benthamiana*	*Nb*MORC1, *Nb*MORC4a, *Nb*MORC4b, *Nb*MORC6a, *Nb*MORC6b, *Nb*MORC7	Manosalva et al., 2015
*Mus musculus*	*Mm*MORC1*, Mm*MORC2a*, Mm*MORC2b*, Mm*MORC3*, Mm*MORC4 *Mm*SmcHD1	Watson et al., 1998; Inoue et al., 1999; Blewitt et al., 2008; Hong et al., 2017
*Homo sapiens*	*Hs*MORC1, *Hs*MORC2, *Hs*MORC3, *Hs*MORC4, *Hs*SMCHD1	Inoue et al., 1999; Kimura et al., 2002; Liggins et al., 2007; Wang et al., 2010; Lemmers et al., 2012

Genome-wide analyses have identified *At*MORC1 homologs in a variety of monocot and dicot plant species (Langen et al., 2014; Table 1). In the cereal barley (*Hordeum vulgare*), the MORC family currently contains seven members. *Hv*MORC1 [HG316119] and *Hv*MORC2 [HG316120], which share 90% aa identity with each other and 47 and 48% aa identity with *At*MORC1, respectively, belong to clade I. *Hv*MORC6a [HG316122] and *Hv*MORC6b [AK372785], which share 38 and 37% aa identity with *At*MORC1, respectively, belong to clade III, and *Hv*MORC7 [HG316121], which exhibits 35% aa identity with *At*MORC1, belongs to clade II (Langen et al., 2014). Through genome wide analysis of the recently published new barley genome assembly (Mascher et al., 2017), two new putative members of the *Hv*MORC family that contain a CW domain have been identified (Figure 1). *Hv*MORCCW1 [HORVU1Hr1G080470.1] and *Hv*MORCCW2 [MLOC_66330.1, HORVU7Hr1G093640.8] share 65% aa sequence identity to each other and 27–29% to *Hs*MORC1-4 family members. A comparison of aa sequence identities for *At*MORC, *H*sMORC, and *Hv*MORC family members is shown in Table 2.

**Table 2:** Amino acid (aa) identity matrix of MORC family members of *Arabidopsis thaliana* (*At*), *Homo sapiens* (*Hs*), and *Hordeum vulgare* (*Hv*).

	***At*MORC1**	***At*MORC2**	***At*MORC3**	***At*MORC4**	***At*MORC5**	***At*MORC6**	***At*MORC7**	***At*GMI1**	***Hv*MORC1**	***Hv*MORC2**	***Hv*MORC6a**	***Hv*MORC6b**	***Hv*MORC7**	***Hv*MorcCW1**	***Hv*MorcCW2**	***Hs*MORC1**	***Hs*MORC2**	***Hs*MORC3**	***Hs*MORC4**	***Hs*SMCHD1**
*At*MORC1	100	80.8	75.6	42.1	40.3	43.2	42.1	17.6	49.2	50.1	42.3	41.5	41.2	23.3	22.0	28.3	30.0	31.0	30.9	19.1
*At*MORC2	80.8	100	80.5	43.9	40.5	43.8	42.7	18.2	48.9	49.9	43.4	42.3	41.8	24.9	24.4	28.9	31.8	30.7	31.7	18.6
*At*MORC3	75.6	80.5	100	41.7	38.4	43.1	40.8	18.8	49.0	49.0	42.3	40.5	40.3	24.8	24.2	28.3	31.9	32.7	31.8	21.2
*At*MORC4	42.1	43.9	41.7	100	49.1	41.2	66.0	21.3	42.3	43.9	41.7	37.2	54.7	22.6	23.7	27.0	27.5	27.8	26.8	15.8
*At*MORC5	40.3	40.5	38.4	49.1	100	38.7	53.6	21.4	41.3	39.3	42.0	37.5	47.9	21.9	23.3	27.4	26.6	27.0	27.9	16.2
*At*MORC6	43.2	43.8	43.1	41.2	38.7	100	41.8	20.8	45.7	46.3	49.2	45.8	38.7	22.9	23.5	28.7	26.6	31.6	31.8	16.2
*At*MORC7	42.1	42.7	40.8	66.0	53.6	41.8	100	20.7	45.5	45.6	43.6	38.9	57.3	22.6	23.6	26.5	27.8	29.6	29.3	16.5
*At*GMI1	17.6	18.2	18.8	21.3	21.4	20.8	20.7	100	18.8	19.3	20.1	17.1	21.7	13.4	12.4	18.9	14.9	14.0	17.1	14.3
*Hv*MORC1	49.2	48.9	49.0	42.3	41.3	45.7	45.5	18.8	100	80.1	43.8	42.2	42.0	24.3	25.6	29.8	27.6	29.8	32.5	18.4
*Hv*MORC2	50.1	49.9	49.0	43.9	39.3	46.3	45.6	19.3	80.1	100	43.9	40.5	41.6	24.1	24.8	27.7	25.1	29.5	32.5	17.9
*Hv*MORC6a	42.3	43.4	42.3	41.7	42.0	49.2	43.6	20.1	43.8	43.9	100	47.9	41.2	24.4	25.7	29.8	30.0	33.9	31.4	17.0
*Hv*MORC6b	41.5	42.3	40.5	37.2	37.5	45.8	38.9	17.1	42.2	40.5	47.9	100	37.2	23.0	23.5	29.9	29.3	31.4	30.9	16.3
*Hv*MORC7	41.2	41.8	40.3	54.7	47.9	38.7	57.3	21.7	42.0	41.6	41.2	37.2	100	22.4	23.9	26.6	26.4	25.9	28.6	16.4
*Hv*MorcCW1	23.3	24.9	24.8	22.6	21.9	22.9	22.6	13.4	24.3	24.1	24.4	23.0	22.4	100	66.9	26.6	28.3	28.7	26.8	15.6
*Hv*MorcCW2	22.0	24.4	24.2	23.7	23.3	23.5	23.6	12.4	25.6	24.8	25.7	23.5	23.9	66.9	100	26.5	29.1	30.9	29.2	15.6
*Hs*MORC1	28.3	28.9	28.3	27.0	27.4	28.7	26.5	18.9	29.8	27.7	29.8	29.9	26.6	26.6	26.5	100	42.7	27.2	27.7	15.4
*Hs*MORC2	30.0	31.8	31.9	27.5	26.6	26.6	27.8	14.9	27.6	25.1	30.0	29.3	26.4	28.3	29.1	42.7	100	29.9	28.2	17.0
*Hs*MORC3	31.0	30.7	32.7	27.8	27.0	31.6	29.6	14.0	29.8	29.5	33.9	31.4	25.9	28.7	30.9	27.2	29.9	100	43.3	17.3
*Hs*MORC4	30.9	31.7	31.8	26.8	27.9	31.8	29.3	17.1	32.5	32.5	31.4	30.9	28.6	26.8	29.2	27.7	28.2	43.3	100	17.5
*Hs*SMCHD1	19.1	18.6	21.2	15.8	16.2	16.2	16.5	14.3	18.4	17.9	17.0	16.3	16.4	15.6	15.6	15.4	17.0	17.3	17.5	100

*aa sequences were aligned using T-Coffee (http://www.ebi.ac.uk/Tools/msa/tcoffee/)*.

Analyses of the solanaceous species tomato, potato, and tobacco have revealed that they contain six *At*MORC1 homologs that are distributed throughout the three phylogenetic clades (Manosalva et al., 2015; Table 1). Based on their aa sequences, the solanaceous MORC proteins share the greatest level of similarity with *At*MORC1, *At*MORC4, *At*MORC6, and *At*MORC7. Notably, potato (*Solanum tuberosum*) *St*MORC1 shares 96% aa identity and 98.5% similarity with tomato (*Solanum lycopersicum*) *Sl*MORC1, with only 12 conservative aa and 15 non-conservative aa differences between them (Manosalva et al., 2015). In addition to these CC-containing MORCs, a gene encoding a CW-containing MORC, *SlMORC3*, was identified in tomato (Langen et al., 2014). The presence of genes encoding both CC- and CW-containing MORCs also has been noted in several other plant species, including rice (*Oryza sativa* ssp. Japonica), soybean (*Glycine max*), and grape (*Vitis vinifera*).

The MORC family in humans is composed of five members. *Hs*MORC1–4 contain both CC and CW domains, in addition to the GHKL ATPase and S5 domains (Li et al., 2013; Hong et al., 2017; Figure 1, Table 1). Note that MORC numbers in animals are not necessarily comparable to those in plants. Based on the domain architecture of CW domain-containing proteins, these MORCs were further divided into two subfamilies. *Hs*MORC1 (984 aa) and *Hs*MORC2 (1,032 aa) comprise subfamily I; they contain a predicted three-stranded CC domain upstream of the CW domain and a two-stranded CC domain at their C-terminus (Perry and Zhao, 2003; Li et al., 2013). *Hs*MORC3 (939 aa) and *Hs*MORC4 (937 aa) belong to subfamily IX; these proteins lack the internal triple-stranded CC domain but contain a predicted two-stranded CC domain downstream of the CW domain. Recently, the CW domains of *Hs*MORC3 and *Hs*MORC4, unlike those of *Hs*MORC1 and *Hs*MORC2, were shown to selectively bind histone H3 peptides trimethylated at lysine 4. The human MORC family also contains a fifth member, SMCHD1 that contains a C-terminal SMC domain linked to the N-terminal GHKL ATPase module (Leong et al., 2013). Sequence analyses have revealed that the MORC family in mouse contains five members with similar architecture to *Hs*MORC1-4 (Hong et al., 2017) as well as SmcHD1, a homolog of SMCHD1 (Blewitt et al., 2008; Table 1).

## MORCs participate in multiple layers of plant immunity

Perception of pathogenic invaders by specific receptors is crucial for the survival of animals and plants. While plants lack the circulating immune cells found in vertebrates, plants and animals both rely on an innate immune system to provide the first line of defense against pathogen attack. In plants, pattern recognition receptors (PRRs) located on the plant cell surface detect the presence of pathogen-/microbe-associated molecular patterns (PAMPs/MAMPs) and trigger PAMP-triggered immunity (PTI) (Martin et al., 2003; Jones and Dangl, 2006; Vlot et al., 2009; Dempsey and Klessig, 2017). In host-pathogen interactions pure PTI is commonly masked by various pathogen effectors, resulting in decreased resistance, called hereafter “basal resistance.” While PTI is frequently sufficient to prevent pathogen colonization, some pathogens have evolved effector proteins that suppress PTI. Plants in turn have evolved R proteins that, following direct or indirect interaction with their cognate pathogen-encoded effector, (also termed an avirulence factor) trigger Effector-Triggered Immunity (ETI; also called *R* gene-mediated resistance). Both PTI and ETI are associated with the activation of immune responses in the inoculated tissue, such as the synthesis of anti-microbial compounds, generation of reactive oxygen species (ROS), expression of defense-associated genes, including *PATHOGENESIS-RELATED* (*PR*)-*1*, and accumulation of the defense signaling hormone SA. In general, these responses are induced more rapidly by ETI than PTI. ETI also is usually associated with the development of a HR, in which necrotic lesions appear at the sites of pathogen entry. Following these events, ETI and PTI can stimulate immune responses in the systemic (uninoculated) portions of the plant, including increased defense gene expression, SA accumulation, and a broad-spectrum, long-lasting resistance called systemic acquired resistance (SAR). In addition to PTI, ETI, and SAR, plants have another layer of resistance, called non-host resistance. Although it is poorly understood, non-host resistance is likely the most common mechanism for protecting plants from the myriad microorganisms they encounter (Mysore and Ryu, 2004). Non-host resistance often involves signaling components and hormones associated with PTI and ETI, suggesting that these forms of resistance overlap.

Analyses of Arabidopsis responding to infection by TCV initially revealed that *At*MORC1 is involved in ETI. Following TCV infection, *atmorc1-1* mutants of the *HRT*-bearing Arabidopsis ecotype Di-17 developed necrosis on the inoculated leaves and supported systemic viral spread, whereas wild type (wt) plants mounted a discrete HR and restricted TCV to the inoculated leaves (Kang et al., 2008; Table 3). Since even greater levels of viral replication and symptom severity were observed when *At*MORC1 and its closest homologs *At*MORC2 and *At*MORC3 were partially silenced, these proteins appear to be functionally redundant. Arabidopsis ecotype Columbia (Col-0) containing knock out (KO) mutations in *AtMORC1* and/or *AtMORC2* also displayed reduced ETI to the bacterial pathogen *Pseudomonas syringae* pv. tomato (*Pst*) carrying the avriulence genes *AvrRpt2* (*Pst AvrRpt2*) or *AvrRpm1* (*Pst AvrRpm1*), and to the oomycete pathogen *Hyaloperonospora arabidopsidis* (*Hpa*) isolates Emco5 (Kang et al., 2010) and Emwa1 (Wang et al., 2011; Table 3). Similar to the results with TCV, growth of *Pst AvrRpt2, Pst AvrRpm1* or *Hpa* Emco5 was substantially greater in the double KO (dKO) line as compared with the single KO lines, although resistance was not as fully compromised as in plants lacking the corresponding *R* genes (Kang et al., 2010). ETI to *Hpa* isolates Hiks1 and Cala2 was not altered in Col-0 plants carrying a single mutation in *At*MORC1 (Wang et al., 2011); however, the lack of a detectable phenotype may be due to the presence of functionally redundant *At*MORCs.

**Table 3:** Immune-related phenotypes of MORC-deficient plants.

**Pathogen/Host**	**MORC homolog**	**Type of resistance**	**Immune phenotype**	**References**
*Turnip crinkle virus/Arabidopsis*	*At*MORC1,2	ETI	Susceptible	Kang et al., 2008
*P. infestans/tomato*	*Sl*MORC1	Basal resistance	Resistant	Manosalva et al., 2015
*P. infestans/potato*	*St*MORC1	Basal resistance	Susceptible	Manosalva et al., 2015
*P. infestans/Nicotiana benthamiana*	*Nb*MORC1	Basal resistance	Resistant	Manosalva et al., 2015
*Pseudomonas syringae/Arabidopsis*	*At*MORC1,2	ETI	Susceptible	Kang et al., 2010
*Hyaloperonospora arabidopsidis/Arabidopsis*	*At*MORC1,2	R-gene mediated resistance	Susceptible	Kang et al., 2010; Wang et al., 2011
*H. arabidopsidis/Arabidopsis*	*At*MORC4,7	ETI	Susceptible	Harris et al., 2016
*Blumeria graminis/barley*	*Hv*MORC1,2,6a,7	ETI	Resistant	Langen et al., 2014
*Fusarium graminearum/barley*	*Hv*MORC1,2	Basal resistance	Resistant	Langen et al., 2014

More recently, *At*MORC4 and *At*MORC7 were shown to play a role in plant defense (Harris et al., 2016; Table 3). While single mutations in AtMORC4 or its closely related homolog *At*MORC7 did not significantly affect ETI to *Hpa* isolate Emwa1, the *atmorc4/atmorc7* double mutant displayed altered expression of many immune response genes and supported increased pathogen growth. *At*MORC7 also appears to be part of a co-expression network consisting of multiple defense genes, including LURP1 (LATE UPREGULATED IN RESPONSE TO *HPA*), PUB12 (PLANT U-BOX 12), ACD6 (ACCELERATED CELL DEATH 6), SDE5 (SILENCING DEFECTIVE 5), and the three NB-LRR type proteins encoded by *At4g12020, At4g36140*, and *At4g36150*.

In addition to ETI, *At*MORC1, and *At*MORC2 have been shown to play a role(s) in several other layers of plant immunity. In comparison to wt Col-0 plants, which are susceptible to TCV due to absence of HRT, the *atmorc1/atmorc2* dKO (Col-0 background) displayed even greater levels of pathogen replication in both the inoculated and systemic leaves (Kang et al., 2012). These dKO plants also exhibited delayed and/or reduced expression of several defense marker genes following virulent *Pst* infection (Bordiya et al., 2016). Moreover, growth of *Pst* and *Hpa* Noco2 were elevated in the dKO and an *atmorc1* single mutant, respectively, as compared with wt plants, indicating that PTI to viral, bacterial and oomycte pathogens was suppressed (Wang et al., 2011; Kang et al., 2012). Consistent with these findings, induction of PTI by treatment with flg22 (a MAMP derived from bacterial flagellin) was partially compromised in the dKO (Kang et al., 2012). Callose deposition and generation of ROS, which are hallmarks of flg22-induced PTI, also were reduced in *atmorc1/atmorc2* dKO plants. Likewise, reduced callose deposition and increased symptom severity were observed when dKO plants were infected with *Phytophthora infestans*, indicating that *At*MORC1 and *At*MORC2 are involved in non-host resistance (Kang et al., 2012). Analysis of SAR revealed that it also was suppressed in the dKO. In comparison to wt plants, primary inoculation of the lower leaves of dKO plants with an avirulent strain of *Pseudomonas syringae* pv. *maculicola* (*Psm*) stimulated less SA accumulation in the systemic leaves, and greater bacterial growth was observed following a secondary inoculation of these upper leaves with virulent *Pst* (Kang et al., 2012).

## Plant MORCs exert opposing effects on immunity in different plant species

Further research into the role of MORC proteins revealed that they positively or negatively affect plant immunity in a species-specific manner (Langen et al., 2014; Manosalva et al., 2015; Table 3). In different barley cultivars, silencing of various *Hv*MORC family members enhanced ETI or basal resistance to the biotrophic powdery mildew fungus *Blumeria graminis* f.sp. *hordei* (*Bgh*), while ectopic over expression of barley MORC proteins compromised *Bgh* resistance. Similarly, silencing *Hv*MORC2 enhanced basal resistance to the necrotrophic fungus *Fusarium graminearum* (Langen et al., 2014; Table 3). Thus, barley MORCs, unlike Arabidopsis MORCs, influence resistance in a negative manner. To determine whether these opposing effects are intrinsic properties of barley and Arabidopsis MORC proteins (which share less than 50% aa identity; Table 2) or are due to differences in their cellular environments, *HvMORC1* and *AtMORC1* were overexpressed in the Arabidopsis *atmorc1/atmorc2* mutant. While *AtMORC1* expression complemented the dKO phenotype by restoring resistance to *Pst AvrRpt2, HvMORC1* expression did not (Langen et al., 2014). *Hv*MORC1's inability to functionally replace *At*MORC1 suggests that differences in the proteins themselves are responsible for their divergent effects on immunity. However, because Arabidopsis and barley are highly divergent plant species, the possibility that Arabidopsis lacks appropriate factors required for *Hv*MORC1 activity cannot be excluded.

MORC1 proteins from the closely related solanaceous species potato, tomato, and tobacco also were found to exert divergent effects on immunity (Manosalva et al., 2015). Although the MORC1 proteins from these three species share >90% aa similarity, analyses of *P. infestans* growth in *MORC1*-silenced plants revealed that potato *St*MORC1, like *At*MORC1, functions positively in immunity, whereas tomato *Sl*MORC1 and tobacco (*N. benthamiana*) *Nb*MORC1, like *Hv*MORC1, negatively influence immunity (Table 3). Consistent with these results, the solanaceous MORC1 proteins exerted different effects in a cell death assay. Transient expression of *St*MORC1 in *N. benthamiana* enhanced cell death induced by the *P. infestans* effector INF1, whereas *Sl*MORC1 and *Nb*MORC1 suppressed it. Domain-swapping studies between *St*MORC1 and *Sl*MORC1 combined with site-directed mutagenesis demonstrated that the contrasting activities of these MORC1 proteins in the cell death assay are determined by just four aa residues in the C-terminal region: three (S/L516, R/G543, and K/E567) reside in the L2 region and one (R/C605) resides in the CC-domain. This finding provides further evidence that the MORC proteins themselves, rather than their cellular environment, are responsible for their species-specific effect on immunity (Manosalva et al., 2015).

## Interacting partners and cellular localization of *At*MORC1 during immune responses

To elucidate *At*MORC1's function in immune responses, its subcellular location was monitored and several interacting partners were identified. Co-immunoprecipitation (Co-IP) analyses revealed that *At*MORC1 interacts with a variety of R proteins that confer resistance to viral, bacterial, oomycete and fungal pathogens (Kang et al., 2008, 2010; Langen et al., 2014; Table 4). Most plant R proteins contain NB and LRR domains coupled to an N-terminal CC domain (CC-NB-LRR) or Toll interleukin-1 receptor domain (TIR-NB-LRR; Martin et al., 2003). *At*MORC1 was initially found to bind HRT, a CC-NB-LRR type R protein, and this interaction was localized to the NB domain (Kang et al., 2008). In addition, *At*MORC1 was shown to physically interact with six other CC-NB-LRR class R proteins from Arabidopsis, a CC-NB-LRR class R protein from barley, two TIR NB-LRR class R proteins from Arabidopsis and the cytoplasmic kinase Pto from tomato (Kang et al., 2008, 2010; Langen et al., 2014; Table 4). Strikingly, the interaction between *At*MORC1 and several R proteins was considerably reduced or undetectable when the R proteins were activated by their cognate effectors, suggesting that *At*MORC1 preferentially binds R proteins in their inactive state (Kang et al., 2010).

**Table 4:** Interaction partners of MORCs (as of July 2017).

**MORC homolog**	**Interactor**	**Targeted pathway**	**Method employed**	**References**
*At*MORC1	HRT	ETI	co-IP	Kang et al., 2008
	RPS2: RESISTANCE TO *Pseudomonas syringae* 2	ETI	co-IP	Kang et al., 2008
	Rx: RESISTANCE AGAINST Potato virus X	ETI	co-IP	Kang et al., 2008
	SSI4: SUPPRESSORS OF NPR1-5-BASED SALICYLIC ACID [SA] INSENSITIVITY	ETI	co-IP	Kang et al., 2008
	RCY1: RESISTANCE TO Cucumber mosaic virus (Y)	ETI	co-IP	Kang et al., 2010
	RPP8: RECOGNITION OF *Peronospora parasitica* 8	ETI	co-IP	Kang et al., 2010
	RPP8c	ETI	co-IP	
	RPM1: RESISTANCE TO *Pseudomonas syringae* pv. *maculicola* 1	ETI	co-IP	Kang et al., 2010
	SNC1: SUPPRESSOR OF NPR1-1, CONSTITUTIVE 1	ETI	co-IP	Kang et al., 2010
	Pto: RESISTANCE AGAINST *Pseudomonas syringae* pv *tomato* [*Pst*]	ETI	co-IP	Kang et al., 2010
	Mla12 (Mildew resistance locus A)	ETI	co-IP	Langen et al., 2014
	HSP90: HEAT SHOCK PROTEIN 90		co-IP	Kang et al., 2010
	FLS2: FLAGELLIN-SENSING 2	PTI	co-IP	Kang et al., 2012
	SUVH2: SUPPRESSOR OF VARIEGATION 3-9 (SU[VAR]3-9) HOMOLOGS	RdDM/Chromatin remodeling	Split-LUC; Y2H	Liu et al., 2014; Jing et al., 2016
	MORC6: MICRORCHIDIA 6	RdDM/ Chromatin remodeling	co-IP-MS; affinity purification-MS; Y2H	Liu et al., 2014; Moissiard et al., 2014
	SWI3B: SWITCH SUBUNIT 3B	Chromatin remodeling	Y2H	Jing et al., 2016
	SWI3C: SWITCH SUBUNIT 3C	Chromatin remodeling	Y2H	Jing et al., 2016
	NAC050: NAC DOMAIN CONTAINING PROTEIN 50	DNA binding	Y2H	Braun et al., 2011
	AT5G11980: CONSERVED OLIGOMERIC GOLGI COMPLEX COMPONENT-RELATED PROTEIN	Golgi transport	Y2H	Braun et al., 2011
*At*MORC6	DMS3: DEFECTIVE IN MERISTEM SILENCING 3	RdDM	Reconstituted Complex	Lorković et al., 2012
	SUVH9: SUPPRESSOR OF VARIEGATION 3-9 (SU[VAR]3-9) HOMOLOGS	RdDM/Chromatin remodeling	co-IP-MS; affinity purification-MS; Y2H; split-LUC	Liu et al., 2014; Jing et al., 2016
	IDN2: INVOLVED IN DE NOVO 2	Chromatin remodeling	Y2H, split-LUC, co-IP	Jing et al., 2016; Liu et al., 2016
	SWI3B: SWITCH SUBUNIT 3B	Chromatin remodeling	Y2H; split-LUC	Jing et al., 2016; Liu et al., 2016
	SWI3C: SWITCH SUBUNIT 3C	Chromatin remodeling	Y2H; split-LUC	Jing et al., 2016; Liu et al., 2016
	SWI3D: SWITCH SUBUNIT 3D	Chromatin remodeling	Affinity purification-MS; split-LUC, co-IP	Liu et al., 2014, 2016
	MORC6: MICRORCHIDIA 6	RdDM/ Chromatin remodeling	Affinity purification-MS	Liu et al., 2014
	MORC1: MICRORCHIDIA 1	RdDM/ Chromatin remodeling	co-IP-MS; affinity purification-MS; Y2H	Liu et al., 2014; Moissiard et al., 2014
	MORC2: MICRORCHIDIA 2	RdDM/ Chromatin remodeling	co-IP-MS; affinity purification-MS; Y2H	Liu et al., 2014; Moissiard et al., 2014
*At*MORC2	SUVH9: SUPPRESSOR OF VARIEGATION 3-9 (SU[VAR]3-9) HOMOLOGS	RdDM/ Chromatin remodeling	Y2H; split LUC	Liu et al., 2014; Jing et al., 2016
	MORC6: MICRORCHIDIA 6	RdDM/ Chromatin remodeling	co-IP-MS; affinity purification-MS; Y2H	Liu et al., 2014; Moissiard et al., 2014
	SWI3C: SWITCH SUBUNIT 3C	Chromatin remodeling	Y2H	Jing et al., 2016
*At*MORC5	UBQ3: POLYUBIQUITIN 3		co-IP-MS	Kim et al., 2013

Whether *At*MORC1 functions in an R protein signaling complex was further assessed by monitoring its ability to interact with RAR1 (REQUIRED FOR MLA12 RESISTANCE 1), SGT1 (SUPPRESSOR OF THE G2 ALLELE of *skp1*), and HSP90. These proteins, which interact with each other, serve as co-chaperones that regulate R protein accumulation and function (Hubert et al., 2009). Co-IPs revealed little to no interaction between *At*MORC1 and either RAR1 or SGT1 and only a weak interaction with HSP90. Thus, *At*MORC1 does not appear to be a component of the HSP90-RAR1-SGT1-R-protein complex (Kang et al., 2010). Consistent with this conclusion, the levels of Myc-tagged resistance protein RPM1 were similar in wt and *atmorc1/atmorc2* dKO plants (Kang et al., 2010), which suggests that *At*MORC1 and *At*MORC2 are not required for R protein stability.

Co-IPs also revealed that *A*tMORC1 interacts with the PRR FLS2 (FLAGELLIN-SENSING 2; Kang et al., 2012; Table 4). FLS2 is a LRR receptor-like serine/threonine-protein kinase that triggers PTI in response to the MAMP flagellin, which is present in a broad range of bacteria (Gómez-Gómez and Boller, 2000; Chinchilla et al., 2006). Unlike the interaction between *At*MORC1 and R proteins, this interaction was not altered in plants treated with flg22 (Kang et al., 2012).

Confocal microscopy and cellular fractionation studies using *N. benthamiana* and *atmorc1/atmorc2* dKO Arabidopsis expressing tagged *At*MORC1 transgenes, respectively, revealed that *At*MORC1 is predominantly localized in endosomes (Kang et al., 2010). Consistent with this finding, a large number of *At*MORC1-associated genes [MAG; originally named CRA (CRT1-associated)] that exhibit altered expression in the dKO line following *Pst AvrRpt2* infection belong to a category designated the “endomembrane system.” Given that *At*MORC1 interacts with FLS2 and that FLS2 co-localizes with endomembrane markers (Lee et al., 2011), it is possible that the interaction between the two proteins occurs in the endosomal compartment. Since some R proteins have been localized to endosomes (Weaver et al., 2006; Kang et al., 2010), this possibility may also apply to certain *At*MORC1-R protein interactions. Thus, *At*MORC1 may affect resistance by playing a role in intracellular trafficking during plant defense.

In addition to endosomes, very low levels of *At*MORC1 were detected in the nuclei of mock-inoculated *atmorc1/atmorc2* dKO Arabidopsis expressing Myc-tagged *At*MORC1 (Kang et al., 2012). Intriguingly, a transient, ~2-fold increase in nuclear *At*MORC1 levels was detected after flg22 treatment or infection with a non-pathogenic PTI-inducing bacterial strain (missing the hrcC cluster for effector secretion), whereas a ~9-fold increase was observed after infection with *Pst AvrRpt2*. These findings suggest that *At*MORC1 undergoes nuclear-cytoplasmic shuttling. Furthermore, since ETI is a stronger immune response than PTI, *At*MORC1 translocation to the nucleus appears to correlate with the strength of the immune response. Analysis of dKO Arabidopsis expressing Myc-tagged *Hv*MORC1 revealed that the barley MORC also accumulated in endomembrane-like vesicles, and the level of nuclear *Hv*MORC1 increased rapidly following flg22 treatment (Langen et al., 2014). *At*MORC1's ability to bind multiple R proteins raises the possibility that it interacts with the NBS region of inactive R proteins in the cytosol and thereby primes them prior to translocation into the nucleus. However, evidence that MORC-interacting R proteins change their subcellular location during defense signaling has not been rigorously established, although RPS4 was shown to be translocated to the nucleus (Wirthmueller et al., 2007).

## Plant MORCs affect transcriptional gene silencing

In addition to localization studies, several other lines of evidence suggested that plant MORC proteins have a nuclear function. For example, *in vitro* assays demonstrated that *At*MORC1 and *Hv*MORC1 bind DNA/RNA and display endonuclease activity (Kang et al., 2012; Langen et al., 2014). The *atmorc1/atmorc2* dKO also displayed greater tolerance than wt plants to the DNA-damaging agent mitomycin C, suggesting that *At*MORC1 and *At*MORC2 serve as negative regulators of DNA repair (Kang et al., 2012). Furthermore, MORC proteins from a range of prokaryotes and eukaryotes have been shown to play roles in chromatin modification and/or DNA recombination and repair (Iyer et al., 2008; Lorković, 2012; Li et al., 2013; Hong et al., 2017).

**Figure 2:**
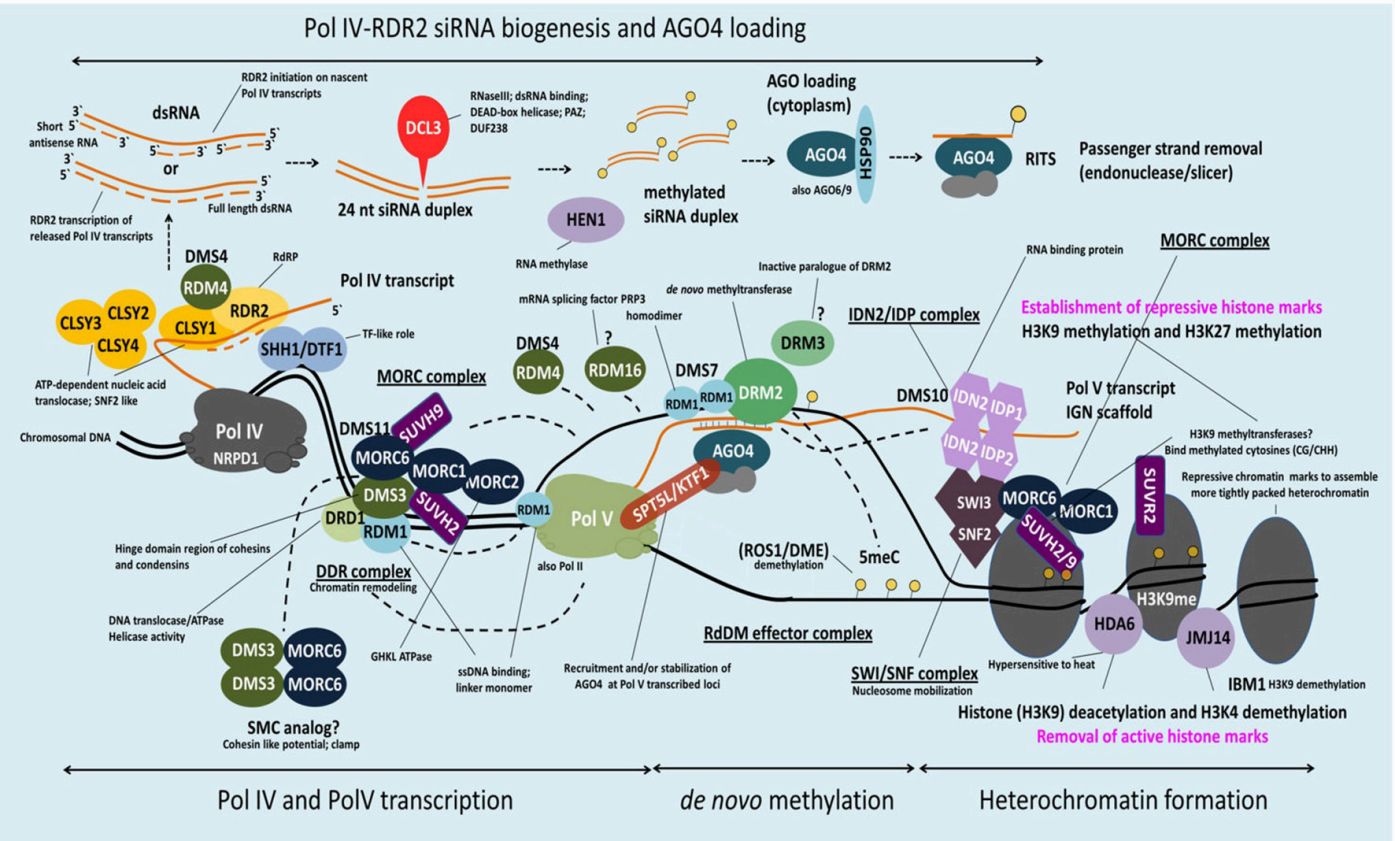
Schematic model of MORC involvement in RdDM. DNA methylation in Arabidopsis is regulated via the RNA-directed DNA methylation (RdDM) pathway. H3K9me2 methylation marks recruit Pol IV to its genomic loci via SHH1 whereas CLSY1 facilitates Pol IV transcription. The single-stranded Pol IV transcripts are converted to dsRNA by RDR2 and subsequently processed by DCL3 in 24-nt siRNAs. Before loading of these siRNAs onto AGO4 (and/or AGO6/9) and export to the cytoplasm they are stabilized by HEN1-mediated 3′ end methylation. AGO4-siRNA complexes are then reimported to the nucleus, where they target in a sequence-specific manner nascent Pol V scaffold transcripts to recruit DRM2 (cytosine-5-methyltransferase), which catalyzes *de novo* methylation at a certain loci. Pol V recruitment to its genomic loci is mediated by the DDR complex. *At*MORC6 together with *At*MORC1 and *At*MORC2 form a second complex (MORC complex), that is thought to be required for the recruitment of Pol V to silenced loci. Therefore, *At*MORC6 is interacting with DMS3, a member of the DDR complex, probably to provide the missing ATPase activity for DMS3. Furthermore, *At*MORC6, *At*MORC1, and *At*MORC2 interact with SUVH2 and/or SUVH9 that act as adaptors to bind methylated DNA and the DDR complex and, in conjunction with *At*MORC proteins, recruit Pol V. Pol V transcripts thereby serve as scaffolds for the assembly of the IDN2-IDP and SWI/SNF chromatin remodeling complexes that adjust nucleosome positioning. The *At*MORC proteins were found to directly interact with IDN2 and/or various subunits of the SWI/SNF complex, thus establishing positioned nucleosomes to effect silencing. Building on this model, it was proposed that binding of methylated DNA by SUVH2 and SUVH9 initially mediates RdDM (via recruitment of the DDR complex and MORC complex) and subsequently facilitates recruitment of a MORC-IDN2-SWI/SNF complex that alters chromatin structure, potentially by positioning nucleosomes at the targeted locus, thereby reinforcing TGS (see text for more information).

The identification of *At*MORC1 and/or *At*MORC6 in three independent forward genetic screens of Arabidopsis mutants defective for transcriptional gene silencing (TGS) provided the first insight into nuclear MORC protein function (Lorković et al., 2012; Moissiard et al., 2012; Brabbs et al., 2013). In plants, TGS plays an important role in repressing transposable elements (TEs), intergenic regions, DNA repeats and some genes; it is mediated by the RNA-directed DNA methylation (RdDM) pathway (Law and Jacobsen, 2010; Figure 2). RdDM utilizes small RNAs to recruit the DNA methylation machinery to targeted sequences. DNA methylation in turn leads to recruitment of histone-modifying enzymes, and the combined effect of these repressive epigenetic marks establishes chromatin in a silenced state. Derepression of silenced reporter genes as well as TEs was observed in *atmorc1* and *atmorc6* mutants, suggesting that these proteins play a role in epigenetic gene silencing (Lorković et al., 2012; Moissiard et al., 2012; Brabbs et al., 2013). Subsequent studies revealed that *At*MORC2, *At*MORC4 and *At*MORC7 also function in TE silencing (Moissiard et al., 2014; Harris et al., 2016).

Despite these findings, the precise role of MORCs in RdDM has not been fully resolved. Analysis of an *atmorc6* mutant (then designated *dms11-1* [W439^*^]) identified in one forward genetic screen revealed a modest reduction in DNA methylation and significant decreases in repressive histone marks that correlated with derepression of a reporter gene and/or certain loci (Lorković, 2012; Lorković et al., 2012). However, *atmorc6* mutants identified in another genetic screen (*atmorc6-5*, C → T transition in codon 41, and *atmorc6-7*, C → T transition in codon 267) exhibited a stochastic silencing phenotype in which the reporter gene, which was derepressed in young plants, became silenced in a cell-autonomous manner in older plants (Brabbs et al., 2013). Since the appearance of TGS correlated with increased levels of DNA methylation, it was proposed that *At*MORC6 promotes efficient RdDM but is not absolutely required for this process. In comparison, analyses of *atmorc1* and *atmorc6* mutants identified by the third genetic screen failed to detect any change in DNA methylation or the repressive histone modification H3K9me2 (dimethylation of histone H3 lysine 9) at either the global level or various up-regulated loci, despite derepression of the silenced reporter gene and several families of TEs (Moissiard et al., 2012). Instead, the nuclei of *atmorc1* and *atmorc6* single and double mutants displayed reduced condensation of pericentromeric heterochromatin. Since the derepressed loci in these mutants largely localized to pericentromeric heterochromatin, it was proposed that *At*MORC1 and *At*MORC6 influence gene silencing downstream of RdDM by modulating higher-order compaction of methylated and silenced chromatin. Consistent with this hypothesis, *At*MORC1 and *At*MORC6 formed small nuclear bodies that were located adjacent to pericentromeric heterochromatin-containing chromocenters. The subsequent demonstration that (i) *At*MORC1 and its closest homolog, *At*MORC2, are functionally redundant for the repression of TEs and protein-coding genes located in heterochromatin, and (ii) *At*MORC1 and *At*MORC2 do not interact with each other but both interact with *At*MORC6, led to the suggestion that *At*MORC6 mediates gene silencing by forming mutually exclusive hetero-dimers with either *At*MORC1 or *At*MORC2 (Liu et al., 2014; Moissiard et al., 2014), or as a homo-dimer (Liu et al., 2014; Table 5).

**Table 5:** Dimerization partners of Arabidopsis MORC proteins.

	**References**
*At*MORC1-*At*MORC6	Liu et al., 2014; Moissiard et al., 2014
*At*MORC2-*At*MORC6	Liu et al., 2014; Moissiard et al., 2014
*At*MORC4-*At*MORC4	Harris et al., 2016
*At*MORC6-*At*MORC6	Liu et al., 2014; Moissiard et al., 2014
*At*MORC7-*At*MORC7	Harris et al., 2016

Efforts to characterize the function of the remaining *At*MORC proteins revealed that *At*MORC4 and *At*MORC7 exclusively form homo-dimers *in vivo* and exhibit partially redundant functions in RdDM (Harris et al., 2016; Table 5). By contrast, *At*MORC5 did not appear to have a significant impact on the transcriptome. In comparison to the *atmorc6-3* single mutant, *atmorc4/atmorc7* double mutant plants differentially expressed 20-fold more loci, with the majority of these showing up-regulation. TEs comprised only 1% of the misregulated loci in *atmorc4/atmorc7*, whereas they constituted 29% of the loci in *atmorc6*. Together, these results suggest that *At*MORC4 and *At*MORC7 predominantly repress the expression of protein-coding genes, whereas *At*MORC6 (and by extension its interacting partners *At*MORC1 and *At*MORC2) preferentially represses TE expression. Further supporting this hypothesis, *At*MORC4 and *At*MORC7 were distributed throughout the nucleoplasm and also present in chromocenter-adjacent bodies, whereas *At*MORC1 and *At*MORC6 generally were detected in punctate foci adjacent to chromocenters (Moissiard et al., 2012; Harris et al., 2016). Analysis of a hextuple *atmorc1/atmorc2/atmorc4/atmorc5/atmorc6/atmorc7* mutant revealed little change in DNA methylation levels at either the global level or at specific up-regulated loci as compared to wt plants (Harris et al., 2016). While this finding suggests that *At*MORCs do not play a major role in RdDM, a small but distinct subset of RdDM loci (~5%) that are poised for transcriptional reactivation did exhibit MORC-dependent methylation changes.

## *At*MORCs interact with components of the RdDM pathway and the SWI/SNF chromatin remodeling complex

Additional clues into MORC proteins' role in TGS have come from the identification of RdDM-associated proteins that directly or indirectly interact with them. *At*MORC6 was initially reported to interact with DMS3 (Figure 2, Table 4), an unusual SMC hinge domain-containing protein that lacks the ATPase motif of GMI1 and SMCHD1 or other known SMC proteins (Lorković et al., 2012). Since DMS3 enhances the ATPase activity of *At*MORC6 *in vitro*, it was proposed that *At*MORC6 provides the missing ATPase activity for DMS3 *in vivo*, thereby generating a functional SMC hinge-containing MORC protein. DMS3 belongs to the DDR complex (composed of DMS3, DRD1 [DEFECTIVE IN RNA-DIRECTED DNA METHYLATION 1], and RDM1 [RNA-DIRECTED DNA METHYLATION 1]), which acts downstream of siRNA production in the RdDM pathway (Law et al., 2010). DDR is required for recruitment of RNA polymerase V (Pol V) to silenced loci and the production of Pol V-synthesized long non-coding RNAs (lncRNAs) that are thought to serve as scaffolds for the assembly of RdDM silencing complexes (Law and Jacobsen, 2010). Consistent with the idea that *At*MORC6 and DMS3 act in a cooperative manner, synthesis of a Pol V-generated transcript was reduced in an *atmorc6* mutant (Lorković et al., 2012).

More recently, *At*MORC1, *At*MORC2 and/or *At*MORC6 were shown to interact with SUPPRESSOR OF VARIEGATION 3-9 (SU[VAR]3-9) homologs SUVH2 and/or SUVH9 (Liu et al., 2014, 2016; Jing et al., 2016) (Figure 2, Table 4). Unlike other SUVH family members, SUVH2 and SUVH9 lack histone methyltransferase activity; however, these proteins contain an N-terminal SRA (SET- and RING-ASSOCIATED) domain capable of binding methylated DNA (Johnson et al., 2014). SUVH2 and SUVH9 play functionally redundant roles in RdDM-mediated gene silencing, as *suvh2*/*suvh9* double mutant plants exhibited greater impairment than the single mutants for DNA methylation at several RdDM loci, production of Pol V-generated lncRNAs, and repression of TEs and various silenced genes (Johnson et al., 2014; Liu et al., 2014). The combined observations that (i) SUVH2 and SUVH9 bind components of the DDR complex but not Pol V (Figure 2), and (ii) the *suvh2/suvh9* mutant displays reduced Pol V-chromatin interaction and DMS3 occupancy at RdDM loci (Johnson et al., 2014; Liu et al., 2014), suggest that SUVH2 and SUVH9 act as adaptors that bind methylated DNA and the DDR complex and, in conjunction with *At*MORC proteins, recruit Pol V. This, in turn, facilitates the production of Pol V-dependent lncRNAs, which promote DNA methylation and thereby create a self-reinforcing loop.

In addition to RdDM, *At*MORC1, *At*MORC2, and/or *At*MORC6, along with SUVH9 and SUVH2, may mediate TGS at some loci via a methylation-independent mechanism. Both *atmorc6-3* and an *suvh2/suvh9* double mutant displayed increased heterochromatin decondensation, altered higher-order chromatin structure, and derepression of some TEs and genes without a corresponding change in DNA methylation levels (Jing et al., 2016). *At*MORC proteins, but not SUVH9, were found to directly interact with the lncRNA-binding protein IDN2 (INVOLVED IN DE NOVO 2) and/or various subunits of the SWI/SNF (SWITCH/SUCROSE NON-FERMENTABLE) chromatin-remodeling complex, including SWI3B, SWI3C, and SWI3D (Liu et al., 2014, 2016; Jing et al., 2016) (Figure 2, Table 4). IDN2 was previously shown to interact with SWI3B and thereby link it to lncRNAs (Zhu et al., 2013). Thus, it was hypothesized that lncRNAs guide ATP-dependent chromatin remodeling complexes to silencing targets, where they establish positioned nucleosomes to effect silencing. Building on this model, it was proposed that binding of methylated DNA by SUVH2 and SUVH9 initially mediates RdDM (via recruitment of the DDR complex) and subsequently facilitates recruitment of a MORC-IDN2-SWI/SNF complex that alters chromatin structure, potentially by positioning nucleosomes at the targeted locus (Zhu et al., 2013), thereby reinforcing TGS (Jing et al., 2016; Liu et al., 2016; Figure 2, Table 4).

## What is the link between MORCs' roles in TGS and immunity?

While a clear mechanistic link between MORCs' role(s) as effectors/modulators of immune responses and epigenetic processes in eukaryotes is current unclear, recent studies have provided important insights. To investigate whether chromatin accessibility is altered in MORC-deficient and pathogen-infected plants, Bordiya et al. (2016) performed DNase I hypersensitive sites sequencing (DNase-seq). Consistent with previous analyses of genome-wide methylation levels (Dowen et al., 2012; Moissiard et al., 2012), neither *Pst* infection nor MORC deficiency substantially altered the genomic distribution of DNase I hypersensitive sites (DHSs). However, pairwise comparisons of DHSs in mock- vs. *Pst*-inoculated plants from the same genetic background (either wt or *atmorc1/atmorc2*) revealed that pathogen infection leads to the appearance of a substantial number of differential DHSs (dDHS); the majority of these were located in genes, although ~20% were located in TEs (TE-dDHS) distributed throughout the genome. Comparisons of DHSs in wt vs. *atmorc1*/*atmorc2* plants receiving the same treatment (either mock or *Pst* inoculation) identified a much smaller number of dDHSs. Strikingly, these *morc1*/*morc2*-enhanced dDHSs were predominantly associated with TEs, particularly TEs located in heterochromatin. ChIP-seq using mock-inoculated *atmorc1*/*atmorc2* plants expressing a myc-tagged AtMORC1 transgene confirmed that *At*MORC1 preferentially binds heterochromatic and/or TE-associated regions. Furthermore, *Pst* infection reduced *At*MORC1 binding at genomic regions that appear to overlap heterochromatic TEs. These results, along with previous studies, argue that *At*MORCs play a critical role in TE repression via interaction with heterochromatin in uninfected plants (Moissiard et al., 2012; Brabbs et al., 2013; Bordiya et al., 2016; Jing et al., 2016).

Analysis of the dDHSs induced by *Pst* infection uncovered a second function of *At*MORC1 as an enhancer of *Pst*-induced gene expression (Bordiya et al., 2016). A previous study noted that SA-induced derepression of TEs generally correlated with increased expression of neighboring protein-coding genes (Dowen et al., 2012). Significantly, many of the *Pst*- induced TE-dDHSs were located proximal to (a)biotic stress-associated genes, including several *PR* and defense genes (Bordiya et al., 2016). Analysis of a subset of these genes showed delayed and/or weaker transcript accumulation in *Pst*-infected *atmorc1*/*atmorc2* as compared to wt plants, suggesting that *At*MORC1 and *At*MORC2 enhance their expression. ChIP-Seq analysis further revealed that *Pst* infection increased *At*MORC1 binding at *Pst*-induced dDHSs found in a small population of euchromatic and heterochromatic TEs and in unannotated, cryptic TEs located within 5 kb upstream of defense genes. Since RNAi-mediated silencing of several *Pst*-induced TE-dDHSs substantially reduced pathogen-induced expression of their neighboring genes, it was suggested that i) these TE-DHSs function as enhancers of proximal defense genes, and ii) activation of these putative enhancers occurs following *Pst*-induced *At*MORC1 and/or *At*MORC2 binding at nearby sites (Bordiya et al., 2016).

To elucidate the enzymatic mechanism(s) through which MORC proteins impact immunity, epigenetic-based gene silencing, and DNA modifications, Manohar et al. (2017) further characterized the DNA-modifying activities of several plant MORC1 proteins. In addition to previously published ATPase and DNA endonuclease activities, these MORC1 proteins were found to exhibit several activities characteristic of type II topoisomerases (topo II), including the ability to (i) covalently bind DNA, (ii) nick/relax and catenate supercoiled DNA, and (iii) decatenate kinetoplast DNA. Like other eukaryotic topo IIs, plant MORC1s were found to contain a short, lysine (K)-rich sequence called a K loop. Mutational analysis demonstrated that the K loop of *Sl*MORC1 is required for DNA-mediated stimulation of ATPase activity and efficient DNA relaxation and catenation activities *in vitro*, and for suppression of INF1-induced cell death *in planta*. However, in contrast to typical topo II enzymes, plant MORC1s appear to require one or more accessory factors present in leaf extracts to complete some of their enzymatic activities, including ATP-dependent, efficient conversion of supercoiled DNA to nicked/relaxed DNA and the formation of topoisomer intermediates. Interestingly, *Sl*MORC1 binds SA, and this suppresses its ATPase and decatenation activities but not its DNA relaxation activity. These findings, combined with *At*MORC1's proposed role in initiating defense gene expression via activation of proximal TE-associated enhancers, suggest that MORC1 proteins may be messengers that translocate to the nucleus in response to *Pst* infection and drive the expression of defense genes by altering the superstructure of TE-associated chromatin.

## Animal MORC proteins in disease regulation

Analyses of human *Hs*MORC2 and *Hs*MORC3 showed that they, like plant MORCs, have topo II-like activities, some of which are suppressed by SA binding (Manohar et al., 2017). Thus, these DNA modifying activities appear to be broadly conserved across two kingdoms. Moreover, all five human MORCs have been associated with cancer. Elevated expression of or mutations in *Hs*MORC1, *Hs*MORC2, and *Hs*MORC4 are linked to breast cancer, multiple myeloma, carcinomas, or B cell lymphoma, while *Hs*MORC3 and SMCHD1 (MORC5) are associated with tumor suppression (Table 6; Li et al., 2013).

**Table 6:** Disease-associated mammalian MORCs.

**MORC species**	**Associated disease/phenotype**	**References**
*Mm*MORC1	Male infertility (mice)	Watson et al., 1998; Inoue et al., 1999
*Hs*MORC1	Psychiatric disorders (depression)	Nieratschker et al., 2014; Schmidt et al., 2016
	Multiple myeloma	Condomines et al., 2007
	Breast cancer	Shah et al., 2009
*Hs*MORC2	Gastric cancer	Tong et al., 2015; Wang et al., 2015; Zhang et al., 2015
	Charcot-Marie-Tooth disease type 2	Zhao et al., 2016
	Lipogenesis (breast cancer)	Sánchez-Solana et al., 2014
*Hs*MORC3	Influenza virus infection	Ver et al., 2015
	Herpes simplex virus	Sloan et al., 2016
	Regulator of cortical bone homeostasis and hematopoietic stem cells niche	Jadhav et al., 2016
	Dermatomyositis	Ichimura et al., 2012; Fiorentino et al., 2013; George et al., 2016
	Down syndrome	Andrews et al., 2016
	Tumor suppression	Andrews et al., 2016
*Hs*MORC4	Inflammatory bowel disease	Söderman et al., 2013
	Chronic pancreatitis	Derikx et al., 2015; Masamune et al., 2015; Norén et al., 2015; Giri et al., 2016; Paliwal et al., 2016
	Lymphoma	Liggins et al., 2007
*Hs*SMCHD1/MmSmcHD1	Facioscapulohumeral muscular dystrophy, X chromosome inactivation	Blewitt et al., 2008; Lemmers et al., 2012
	Tumor suppression	Leong et al., 2013

Similar to plant MORC proteins, animal MORCs have been implicated in gene silencing. Derepression of a silenced reporter gene was observed in *Caenorhabditis elegans* deficient for MORC1, the only MORC homolog present in this organism (Moissiard et al., 2012). More recently, *Ce*MORC1 was proposed to function as a link between the germline nuclear RNAi pathway and transgenerational silencing via its role in maintaining both repressive epigenetic marks and heterochromatin compaction at targeted loci (Weiser et al., 2017). In mice, a mutation in *MmMORC1* conferred reduced DNA methylation of specific classes of TEs, which led to compromised TE repression in the male germline (Pastor et al., 2014), while SmcHD1 is required for the maintenance of X chromosome inactivation and hyper-methylation of CpG islands (Blewitt et al., 2008). *Hs*MORC2 and *Hs*SMCHD1 also play important roles in epigenetic gene silencing. *Hs*MORC2 down-regulates *Carbonic Anhydrase IX* (*CAIX*) expression in tumor cells by recruiting histone deacetylase 4 to the *CAIX* promoter, which in turn alters histone acetylation levels (Shao et al., 2010), while SMCHD1 is involved in silencing the D4Z4 metastable epiallele whose overexpression leads to facioscapulohumeral muscular dystrophy type 2 (Lemmers et al., 2012). In addition, the human *Hs*MORC3 (syn. NUCLEAR MATRIX PROTEIN 2; NXP2) binds SUMO2 (SMALL UBIQUITIN-LIKE MODIFIER 2) to promote gene silencing (Rosendorff et al., 2006).

Localization studies have shown that mammalian MORC3 is localized to PROMYELOCYTIC LEUKEMIA PROTEIN (PML) nuclear bodies (PML-NB; Mimura et al., 2010); this is reminiscent of plant MORCs, which form nuclear bodies adjacent to chromocenters (Moissiard et al., 2012; Harris et al., 2016). MORC3 also is associated with replication of influenza virus and herpes simplex virus 1 (HSV-1). It binds to the influenza viral polymerase, co-localizes with viral ribonucleoproteins, and likely regulates transcription at the epigenetic level to modulate viral RNA replication (Ver et al., 2015). Its antiviral role is further supported by a recent study demonstrating that MORC3 is recruited to sites associated with HSV-1 genomes after their entry into the nucleus of infected cells (Sloan et al., 2016; Table 6).

## Conclusion and future research

Mounting evidence indicates that plant and animal MORC proteins play critical roles in gene silencing and disease progression. However, while recent studies have begun to provide some insights into how members of this evolutionarily conserved protein family exert their effects, much remains unknown. In both plants and animals, the MORC family contains multiple members, with the single *MORC* gene in *C. elegans* a notable exception (Simpson et al., 1986). Besides the GHKL fold and 5S domains, the aa sequences of plant and human MORCs exhibit little conservation (Manohar et al., 2017). However, some similarities in domain architecture have been noted, including (i) a C-terminal CC that is found in most plant and animal MORCs (Inoue et al., 1999; Perry and Zhao, 2003; Li et al., 2013; Langen et al., 2014; Manosalva et al., 2015), (ii) CW domains, which are present in human HsMORC1-4 and a subset of plant MORCs (Langen et al., 2014), and (iii) an SMC hinge domain, which has been identified in divergent members of the MORC family in animals and Arabidopsis (Blewitt et al., 2008; Böhmdorfer et al., 2011; Leong et al., 2013). Studies of plant MORCs have primarily focused on the CC-containing subgroup and their role in TGS and immunity. In comparison, limited analysis of GMI1 suggests that it is involved in DNA double-strand break repair (Böhmdorfer et al., 2011), and the function of the CW-containing MORCs is completely unknown. Future efforts to characterize MORC proteins will require not only deciphering the function of various MORC protein domains, but also determining the activities of MORC family members with different domain architectures.

Due to the absence of a clear correlation between TE/gene derepression and changes in DNA methylation patterns in Arabidopsis *atmorc* mutants, *At*MORCs' role in TGS via RdDM vs. through a DNA methylation-independent mechanism has been a topic of debate. However, the recent discovery that *At*MORCs interact with components of both the RdDM-pathway and the SWI/SNF chromatin remodeling complex provides a potential explanation that could reconcile these conflicting possibilities (Jing et al., 2016; Liu et al., 2016). Future efforts to gain a better understanding of how TGS is mediated at specific loci will require clarifying the function of *At*MORC proteins in the RdDM and SWI/SNF chromatin remodeling complexes, identifying other *At*MORC-interacting proteins, and determining how these complexes interface with each other to effect silencing.

Plant MORC proteins also have been implicated in multiple layers of immunity. The discovery that *At*MORC1 binds a wide variety of R proteins and the PRR FLS2, and that the MORC-R protein interaction is disrupted by R protein activation, provides a small clue into how ETI and PTI are influenced by MORC proteins (Kang et al., 2008, 2010, 2012; Langen et al., 2014). However, many questions remain, including (i) how does disruption of the MORC-R protein interaction impact resistance signaling, (ii) what mechanism is responsible for disrupting this interaction, (iii) how do MORC proteins influence SAR and non-host resistance, and (iv) do MORCs have additional functions in the endosome? In addition, the mechanism through which MORC1 proteins from different species exert a positive or a negative influence on disease resistance remains unclear. Mutational analyses suggest that these species-specific effects are an inherent function of the MORC1 proteins themselves, rather than their cellular environment (Manosalva et al., 2015). Given that the C-terminal region of recombinant *St*MORC1 and *Sl*MORC1 is required for homo-dimerization and this region of *Sl*MORC1 is phosphorylated *in vitro*, additional studies are required to evaluate whether phosphorylation of MORC1's C-terminus influences its ability to dimerize and/or interact with other proteins, potentially including R proteins and/or other positive or negative regulators of immunity.

Although the link between MORCs' roles in immunity and TGS is currently unknown, the discovery that *Pst* infection alters *At*MORC1 binding at genomic regions preferentially associated with TEs provides an important clue. A growing number of studies suggest that TEs are key regulatory elements that control stress-associated gene expression (Dowen et al., 2012). Thus, the discovery that *Pst* infection reduces *At*MORC1 binding at loci associated with heterochromatic TEs led Bordiya et al. (2016) to propose that loss of *At*MORC1 binding at these sites disrupts a silencing complex and thus upregulates heterochromatic TE expression. Concurrently, *Pst*-enhanced binding of *At*MORC1 at sites near defense gene-associated TEs might alter associated protein complexes and thereby temporarily relieve silencing; the derepressed TEs could then serve as enhancers of proximal gene expression. An alternative, although not mutually exclusive, possibility is that the topo II-like DNA-modifying activities of plant MORC1 proteins directly impact gene silencing and defense gene activation (Manohar et al., 2017). The DNA modifying activities of MORCs may be regulated by the RdDM pathway and/or by the SWI/SNF chromatin remodeling complex; note that *Sl*MORC1 only displayed efficient, ATP-dependent DNA modifying activity in the presence of a tomato extract, which suggests that an additional factor(s) is required for full activity. Therefore, identifying the cellular contexts through which the stability and/or enzymatic activities of these complexes is altered should provide valuable insights into MORC1's mechanism(s) of action.

It is becoming increasingly clear that animal MORCs share many similarities with their plant counterparts. Indeed, all five members of the human MORC family have been linked to disease development or tumor suppression. In addition, several animal MORCs have been implicated in epigenetic gene silencing. Animal MORCs also exhibit topo II-like DNA modifying activities. This finding, combined with the observations that (i) SA suppresses some DNA modifying activities of animal and plant MORCs, and (ii) SA is an important regulator of inflammation in animals and immune responses in plants, raises the possibility that future studies will uncover similarities in the mechanism(s) and regulatory processes that govern how MORCs from both kingdoms mediate at least some of their effects.

## Author contributions

AK: wrote the paper and designed the figures. HK: wrote the paper. JS: wrote the paper and designed the figures. DD: wrote the paper. DK: wrote the paper. KK: wrote the paper.

### Conflict of interest statement

The authors declare that the research was conducted in the absence of any commercial or financial relationships that could be construed as a potential conflict of interest.
